# Healthcare provider perspectives on a clinical decision tool to support individualized exercise prescriptions and discussions for breast cancer survivors

**DOI:** 10.1007/s11764-025-01750-3

**Published:** 2025-03-12

**Authors:** Jinani Jayasekera, Oliver W. A. Wilson, Kaitlyn M. Wojcik, Eleanor M. Kerr, Rachelle Brick, David Berrigan, Jennifer Yeong-shin Sheng, Takeo Fujii, Kathleen Thomas, Henri K. Parson, Padma Sheila Rajagopal, Richard L. Street

**Affiliations:** 1 National Institute On Minority Health and Health Disparities, Intramural Research Program, National Institutes of Health, Bethesda, MD 20892, USA; 2 Ripple Effect Communications, Inc, Rockville, MD, USA; 3 Women’s Malignancies Branch of the Center for Cancer Research at the National Cancer Institute, National Institutes of Health, Bethesda, MD, USA; 4 Department of Oncology at the Johns Hopkins University School of Medicine, Baltimore, MD, USA; 5 Health Systems and Interventions Research Branch of the Healthcare Delivery Research Program in the Division of Cancer Control & Population Sciences at the National Cancer Institute, National Institutes of Health, Rockville, MD, USA; 6 Division of Cancer Control & Population Sciences at the National Cancer Institute, National Institutes of Health, Rockville, MD, USA; 7 Department of Health, Physical Education, Exercise Science, Norfolk State University, Norfolk, VA, USA; 8 Macon and Joan Brock Virginia Health Sciences at Old Dominion University, Eastern Virginia Medical School, Norfolk, VA, USA; 9 Cancer Data Science Laboratory in the Center for Cancer Research at the National Cancer Institute, National Institutes of Health, Bethesda, MD, USA; 10 Department of Communication and Journalism at Texas A&M University, College Station, TX, USA

**Keywords:** Breast cancer survivors, Exercise prescriptions, Exercise discussions, Clinical decision tools

## Abstract

**Purpose:**

We evaluated healthcare providers’ current knowledge, practices, and perspectives on a novel clinical decision tool (beta-version) to facilitate individualized exercise prescriptions and discussions in clinical settings.

**Methods:**

We recruited healthcare providers who had treated or provided care to breast cancer survivors aged ≥ 35-years in the past 12 months. The participants were presented with a tool to provide individualized exercise recommendations considering women’s individual, clinical, and contextual characteristics. Validated and reliable pre-existing instruments were used to survey providers’ current knowledge, practices regarding exercise discussions, and perspectives on the beta-version (paper-draft) of the novel tool.

**Results:**

The sample consisted of complete survey responses from 177 healthcare providers including breast oncologists (27.7%), primary care physicians (10.7%), exercise specialists (19.8%), occupational/physical therapists (18.1%), advanced care providers, nurses, navigators, and social workers (23.7%). Median years of experience was 8-years (range: 5–13). Overall, 62.1% (*n* = 110) reported that they were knowledgeable about counseling survivors based on exercise guidelines. Among breast oncologists and primary care physicians (*n* = 68), only 39.7% reported that they were knowledgeable about identifying patients for exercise referals. The majority agreed that they would find the tool offering individualized information useful (*n* = 148, 83.6%), and would use it regularly to inform practice (82.5%). ‘Exercise Readiness’, ‘Exercise Resources at Home’, and ‘Quality-of-Life’ were the highest rated items for inclusion in the tool for exercise prescriptions. Provider perspectives were incorporated into the beta-version of the tool.

**Conclusion:**

A clinical decision tool considering individual, clinical, and contextual characteristics may support exercise prescriptions and discussions in clinical settings.

**Implications for cancer survivors:**

An evidence-based tool for exercise prescriptions may increase healthcare provider confidence to discuss, educate, encourage, and provide exercise referrals for breast cancer survivors.

## Introduction

There are over four million breast cancer survivors currently living in the U.S. [1]. While most women diagnosed with breast cancer are likely to experience long-term survival, they are also at increased risk of treatment-related side-effects and poor quality-of-life [2]. Exercise may offer various clinical benefits to survivors, including substantial improvements in survival, quality of life, physical function, and adverse treatment-related outcomes [3-5]. However, most women diagnosed with breast cancer may not experience exercise benefits due to difficulties in engaging in exercise following treatment. National level data suggest that less than 40% of the women diagnosed with breast cancer engage in guideline-recommended levels of aerobic exercise [6]. Survivors report breast cancer–related lymphedema, fatigue, pain, discomfort, lack of access to specialized services, and competing needs as major barriers to exercise participation [7, 8].

Healthcare providers could play a critical role in improving exercise participation among cancer survivors [9]. Studies show that patients prefer receiving information about exercise from their healthcare providers, and patient–provider discussions could help encourage patients to exercise [10-12]. While most providers agree that it is important to discuss exercise with patients, they also report lack of time, low confidence, and safety concerns as barriers to these discussions [13]. Currently, healthcare providers have limited tools and resources targeted to their needs to help overcome these barriers and support exercise discussions in clinical settings [14].

Current cancer exercise guidelines recommend clinicians provide individualized ‘exercise prescriptions’ for cancer survivors [15-18]. However, the guidelines themselves provide limited information on the optimal frequency, intensity, duration, and type of exercise a healthcare provider may need to consider in an exercise prescription given the survivor’s individual characteristics. This is important as studies suggest that individual demographic (e.g., age), clinical (e.g., comorbidities), and contextual (e.g., neighborhood safety) factors could determine exercise participation and health outcomes in breast cancer survivors [19-22]. Moreover, individualized exercise prescriptions may require healthcare providers to conduct comprehensive assessments of needs, values, and preferences of women diagnosed with breast cancer. However, it is unclear how ‘exercise prescriptions’ would be implemented practically as a part of survivorship care.

To address these knowledge gaps, we evaluated healthcare providers’ current knowledge and practices regarding exercise discussions with breast cancer survivors. We also gathered provider perspectives on a beta-version of a clinical decision tool that could help support individualized exercise prescriptions and discussions in practice. Finally, we examined any differences in knowledge, practices, and perspectives on the tool across provider characteristics (e.g., urban–rural practice location). The overarching goal of this study was to inform the development of an individualized clinical decision tool to support exercise prescriptions and discussions for breast cancer survivors in clinical settings.

## Methods

### Development of a beta-version of the clinical decision tool

The design of the clinical decision tool was guided by the Collaborative Deliberation Model [23], which has been used in previous studies to inform the development of tools for shared decision-making in clinical practice [24-26]. The model provides five components for consideration in tool development, which includes ‘Constructive Engagement’, ‘Choice Recognition’, ‘Learning’, ‘Preference Elicitation’, and ‘Integration’. For this study, a paper-draft of the clinical decision tool was developed to gather healthcare provider perspectives on the tool.

Accordingly, we first identified items deemed necessary for a clinical decision tool to facilitate ‘Constructive Engagement’ about exercise in a clinical setting. The items were identified based on the current literature and expertise of a team of physicians (*n* = 3), exercise specialists (*n* = 4), breast cancer survivors (*n* = 5), and tool developers (*n* = 1). The items included individual (e.g., age), clinical (e.g., stage at diagnosis, treatment), and contextual determinants of exercise (e.g., residential greenness); the potential individualized benefits of exercise (e.g., improvements in quality-of-life, reduction in the risk of breast cancer death); and clinical conditions for consideration during an exercise discussion (e.g., lymphedema).

For ‘Choice Recognition’, the tool used the breast cancer survivor’s individual (e.g., age) and clinical (e.g., stage) characteristics to provide individualized estimates of breast cancer outcomes (e.g., breast cancer death) associated with meeting exercise guidelines [18, 27], less than meeting guidelines, and no/minimal exercise. Women were considered to have ‘met aerobic guidelines’ if they participated in an equivalent of ≥ 150 min per week of moderate-intensity physical activity; and ‘met muscle strengthening guidelines’ if they participated in ≥ 2 days per week of muscle-strengthening exercise [18, 27]. We also noted in the paper-draft of the tool that the individualized estimates were generated by an established simulation (mathematical) model that has been used previously to develop ‘calculation engines’ for clinical decision tools [26, 28, 29]. Accordingly, healthcare providers could use the tool to support exercise prescriptions and discussions using model-based individualized breast cancer outcomes in addition to the individual characteristics reported in the tool. For example, the tool could provide 10-year breast cancer specific survival rates of 86% for minimal exercise (i.e., < 30 min/week of aerobic exercise); 88% for 30–60 min/week of aerobic exercise (i.e., less than meeting guidelines); and 91% for meeting aerobic exercise guidelines for a 55-year-old woman diagnosed with stage I, hormone receptor positive breast cancer, who had undergone surgery, currently on endocrine therapy, and has no other conditions preventing exercise. The healthcare provider may use this information to discuss the absolute benefits of exercise and recommend progressing towards meeting exercise guidelines considering the woman’s individual characteristics. The ‘exercise prescription’ may also include any contextual determinants selected by a woman for the healthcare provider to consider in a discussion. For example, the tool included items such as access to healthy food and exercise facilities.

The paper-draft of the tool (see Data Supplement, Beta-version of the Clinical Decision Tool) was presented to the healthcare providers during cognitive interviews and survey as described below. Healthcare providers were invited to suggest additional items necessary for inclusion in the tool.

### Survey development

Items were adapted from pre-existing instruments. A detailed description is available in the Supplementary Material (Data Supplement, Survey Items). Cognitive interviews were conducted with 10 healthcare providers to assess their understanding of the survey questions and solicit input on any questions or concepts they considered missing or needing revision. Feedback from the interviews were used to further refine the survey and the beta-version (paper-draft) of the clinical decision tool. For instance, participants suggested adding more tool input characteristics (e.g., historical treatment), and conditions (e.g., nutritional deficiencies) to the survey.

### Participants

We aimed to recruit 150 healthcare providers using convenience and snowball sampling. Breast oncologists, primary care providers, nurse practitioners, nurses, social workers, patient navigators, exercise specialists, and occupational and physical therapists, who had treated or provided care to a breast cancer survivor (i.e., women diagnosed with breast cancer) aged ≥ 35-years in the past 12 months (June 2023-June 2024) were recruited for the study. Participants were recruited by an external contractor via professional organizations (e.g., the American Society of Clinical Oncology (ASCO)) and publicly available information. All participants provided informed consent and received $125-$150 for each cognitive interview (i.e., for survey development described above), and $60–75 for completing the survey. Cognitive interview participants (*n* = 10) were excluded from the survey. The study was approved by the National Institutes of Health Institutional Review Board and was considered as exempt research based on the use of de-identified data.

## Measures

### Healthcare provider and practice characteristics

The survey collected information on age (years), length of professional experience (years), type of healthcare profession(s) [30], race [31], ethnicity [31], exercise training/education (formal and/or continuing), and exercise participation [32] using validated and established instruments (Data Supplement, Survey Items).

Information on the type of practice [9] (e.g., community) and rural/urban location [33] were collected. Healthcare providers (except exercise specialists) were asked if they had the ability to provide a referral to an exercise specialist. Providers were also asked to report the proportion of breast cancer survivors seen by, 1) phase in the cancer care continuum (e.g., post-treatment), 2) meeting exercise recommendations (aerobic (≥ 150 moderate-intensity min/week), muscle strengthening (≥ 2 days/week)) [17, 18], and 3) medically or physically safe to engage in exercise.

### Healthcare provider knowledge and discussions about exercise

Provider knowledge and current practices concerning the provision of exercise advice were measured using five-point bipolar Likert scales with items adapted from the ‘Clinicians Perspectives on Exercise in Patients with Cancer’ (CliPEC) questionnaire [30]. Providers were asked to rate their knowledge on counseling breast cancer survivors according to current cancer exercise guidelines. Characteristics of exercise discussions (e.g., role of exercise in symptom management) were rated on a likelihood scale. Knowledge on referrals were only rated by healthcare providers (e.g., physicians) with the ability to refer patients to exercise programs. Other resources/tools used by providers to support exercise discussions were collected via open-ended questions.

### Healthcare provider perspectives on the beta-version of the clinical decision tool

Healthcare providers were presented with the beta-version (paper-draft) of the novel tool (Data Supplement, Beta version of the Clinical Decision Tool). Providers were asked to rate the usefulness of the tool, likelihood of using it on a regular basis, and if using the tool would increase their confidence in discussing exercise with breast cancer survivors. They were also asked about a preferred format (e.g., mobile), and when the tool should be accessible to patients (e.g., before a consultation) [34]. Providers who agreed that the tool will be useful in clinical settings were asked to rate the usefulness of the tool to educate, encourage, facilitate shared decision-making, identify resources, and refer patients to exercise professionals. Healthcare provider suggestions to make the tool more useful and increase confidence were collected via open-ended questions.

### Healthcare provider perspectives on tool characteristics

Using a five-point bipolar Likert scale, the providers were asked to rate the extent to which they agreed or disagreed on the functionalities and specific characteristics to include in the tool to generate individualized exercise recommendations. The inputs were individual demographic (e.g., age), clinical (e.g., stage at diagnosis), and contextual (e.g., residential greenness) characteristics. Providers were also asked to rate the potential individualized benefits (e.g., quality-of-life) of exercise, and conditions associated with breast cancer and treatment they would consider for the tool. Finally, they were asked to rate outputs (e.g., referral suggestions) generated by the tool. Additional items for tool inclusion were collected via open-ended questions.

## Analyses

We calculated percent agreement and 95% confidence intervals for each survey question. A ‘missing’ category was created for missing values. Healthcare providers’ knowledge, discussions, and perspectives on the tool were compared by provider type and practice location. The providers were grouped into three types including, 1) breast oncologists and primary care physicians, who may counsel and/or make exercise referrals, 2) exercise specialists, who may receive referrals (exercise physiologists, exercise trainers/specialists, physical and occupational therapists), and 3) advanced care providers, navigators, social workers, and nurses who may counsel and/or make referrals depending on the healthcare system. Provider practice location was grouped into rural and suburban/urban locations.

Thematic analysis was conducted to identify the dominant themes and sub-themes in participants’ responses to the open-ended survey questions [35]. Qualitative data from the open-ended questions were reviewed and descriptively coded by two researchers (OW; KW) independently. The constant comparison method was used to continually revaluate and revise the codebook during analysis [36]. Disagreements were resolved via a consensus discussion process and triangulation with emerging evidence. Multiple trained investigators knowledgeable about exercise (OW; HP; KT; RB; DB), breast cancer (TF; JS; PSR), clinical decision tools (JJ), communication (RS; EK), and health equity (JJ; KT; HP) reviewed the analyses [37]. Descriptive statistics were computed using STATA (version 18.0) and Microsoft Excel.

## Results

### Healthcare provider and practice characteristics

Complete survey responses were collected from 177 individual healthcare providers. The median age of the sample was 43 years (Standard Deviation (SD): 16; range 34–53), and the majority were female (67.2%) (Table 1). According to self-reported race and ethnicity, 8.5% were Asian, 19.8% non-Hispanic Black, and 60.5% non-Hispanic White. The sample included breast oncologists (27.7%), primary care physicians (10.7%), exercise specialists (19.8%), and occupational/physical therapists (18.1%). The median years of professional experience was eight years (SD: 8; range 5–13). Most providers worked at Cancer Centers (37.9%), and only 8.5% reported working in a rural location.

On average, providers reported that 55% of the breast cancer survivors seen in their practice were on- or post-treatment, and 74–75% of the women were medically and/or physically safe to engage in exercise. Nearly half of the providers reported receiving no/minimal formal (46.9%) or continuing (48.6%) exercise education (Data Supplement, Education). There were missing values for healthcare provider’s formal education (*n* = 9, 5.1%) and continuing education (*n* = 2, 1.1%).

### Healthcare provider knowledge and discussions about exercise

Overall, 62.1% (*n* = 110) of the providers reported that they were knowledgeable about how to counsel breast cancer survivors based on current exercise guidelines (Table 2). The percentage knowledgeable on how and when to counsel women based on current exercise guidelines were higher among exercise specialists (how: 86.6%; 95% Confidence Interval (CI) 77.8–95.3%; when: 80.6%; 95%CI: 70.1–91.1%) compared to breast oncologists and primary care physicians (how: 55.9%; 95%CI: 40.1–71.7%; when: 45.6%; 95%CI: 28.1–63.1%) or advanced care providers, nurses, navigators, and social workers (how and when: 33.3%; 95%CI: 8.6–58.0%) (Table 2).

Only 39.7% (95%CI: 21.3–58.2%) of the breast oncologists and primary care physicians reported that they were knowledgeable about identifying patients to refer to an exercise program (Table 2). Over 30% (*n* = 59) of the providers indicated that they were currently not using any resources or tools to discuss exercise with breast cancer survivors, and over 10% (*n* = 20, 11.3%) had missing values for this item (Data Supplement, Resources).

### Healthcare provider perspectives on the clinical decision tool (beta-version)

Most providers agreed that they would find a tool offering individualized information useful (*n* = 148, 83.6%) and would use it regularly to inform clinical practice (*n* = 146, 82.5%) (Table 3). Compared to breast oncologists and primary care physicians (72.1%; 95%CI: 59.5–84.6%), a higher percentage of patient navigators, social workers, and nurses agreed that the tool would be useful (92.9%; 95%CI: 84.8–100%). The percentage of breast oncologists and primary care physicians who agreed that the tool would increase their confidence to talk about exercise with patients was 83.8% (95%CI: 74.3–93.4%). In open-ended questions, providers reported that the tool would contribute to their confidence by providing access to evidence-based information (*n* = 33), discussion guidance (*n* = 22), offering individualized information (*n* = 18), increasing knowledge (*n* = 8), and improving clinical workflow efficiency (*n* = 4) (Data Supplement, Additional Feedback).

The providers preferred a mobile (*n* = 154, 87.0%) or web-based (*n* = 140, 79.1%) application of the tool. A higher proportion of breast oncologists and primary care physicians (80.9%; 95%CI: 70.5–91.3%) and exercise specialists (83.6%; 95%CI: 73.9–93.3%) agreed that the tool should be used during a clinical consultation with their patients. Two participants indicated that the tool should be available for use “with an exercise physiologist”, and “at all times because patients vary in their readiness to receive this information”.

Among those who agreed that the tool would be useful in practice (*n* = 148), 91.9% (*n* = 136) reported that they would use the tool to encourage women to engage in exercise, and 83.1% (*n* = 123) would use it to facilitate shared decision-making about exercise in clinical settings. In open-ended questions, providers suggested adding a community forum (*n* = 14), and educational resources (*n* = 9) to make the tool more useful.

### Healthcare provider perspectives on tool characteristics

#### Inputs

**Fig. 1:**
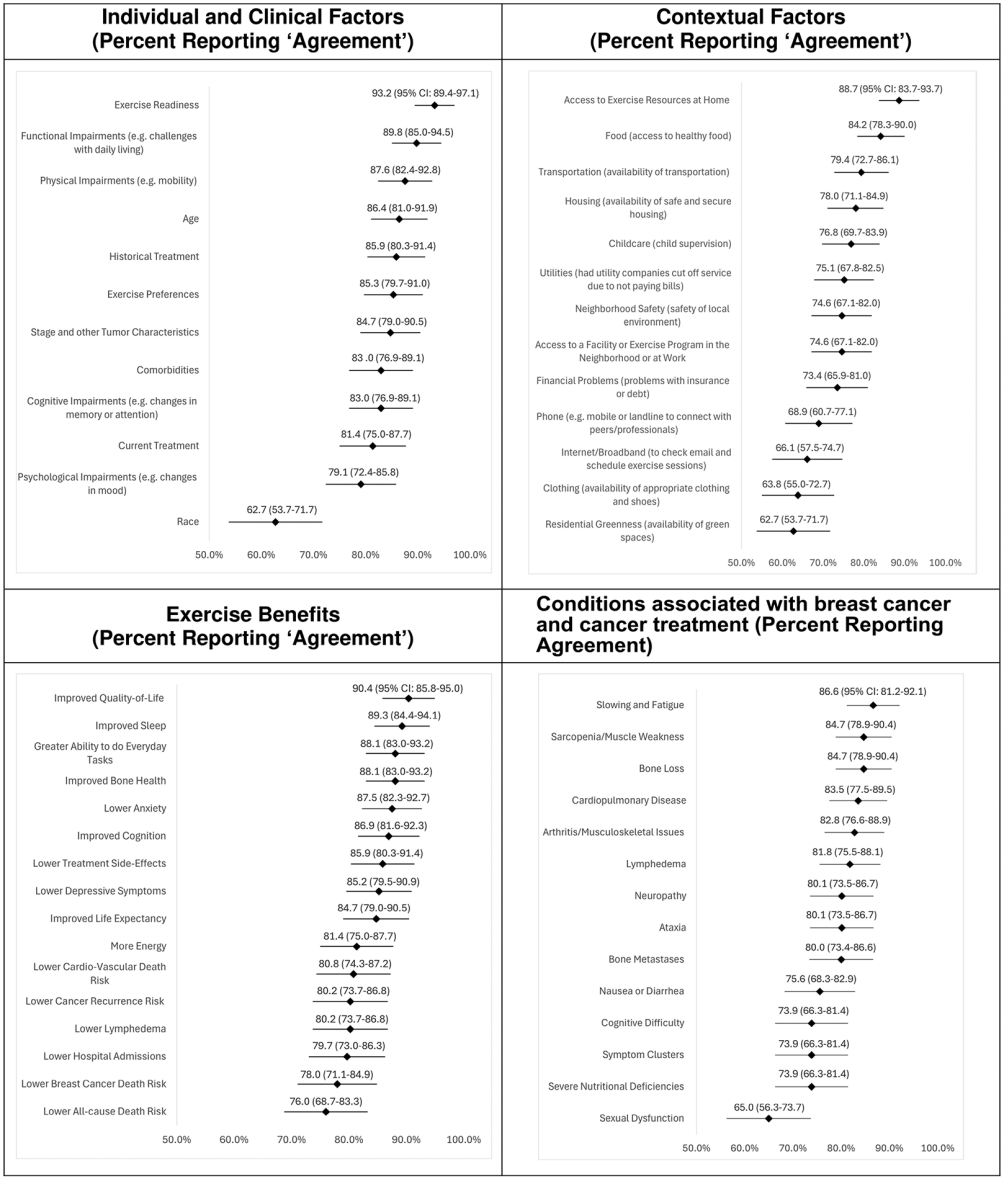
Healthcare provider perspectives on clinical, demographic, contextual, exercise benefits, and risk factors to consider in a clinical decision tool for individualized exercise prescriptions and discussions

Providers agreed that a tool providing individualized exercise recommendations should consider a woman’s readiness to exercise (93.2%), functional (89.8%) and physical (87.6%) impairments, and age (86.4%) (Fig. 1). Providers also agreed that the tool should consider the availability of exercise resources at home (88.7%), and access to food (84.2%). Inputs that received a lower rating (< 70%) were race, ethnicity, access to phone, internet, clothing and green spaces.

#### Benefits

Over 90% (95%CI: 85.8%–95.0%) agreed that the tool should include ‘improved quality-of-life’ as an exercise benefit (Fig. 1). Other benefits mentioned in the open-ended questions included improved strength and reduced risk of sarcopenia.

#### Conditions associated with breast cancer and cancer treatment

Over 86% of the providers agreed ‘slowing and fatigue’ should be integrated into the tool (Fig. 1). Other conditions mentioned were impaired range of motion, cardiotoxicity, and stress.

#### Tool output

Approximately 86% (95%CI: 80.3%–91.4%) of the providers agreed that output from the tool could include ‘exercise referral suggestions’, while 80.2% (95%CI: 73.7%–86.8%) and 78.5% (95%CI: 71.7%–85.4%) agreed that tool output could include individualized benefits and risks associated with aerobic and muscle strengthening exercise, respectively.

### Comparisons across practice locations (rural, suburban, urban)

The percentage of healthcare providers who agreed that they had the knowledge to counsel women based on current exercise guidelines was similar across the practice locations (Data Supplement, Knowledge and Discussions by Practice Location). However, the point estimate for the percentage of providers agreeing that they knew when to counsel women about exercise was lower in rural (40.0%; 95%CI: 0.8–79.2%) compared to urban/suburban (57.4%; 95%CI: 47.4–67.5%) locations.

All providers practicing in rural locations (*n* = 15) agreed that the tool would be useful to discuss exercise with breast cancer survivors (Data Supplement, Tool Perspectives by Practice Location). The proportion of providers agreeing that the tool would be useful to encourage women to engage in exercise was 86.7% (95%CI: 68.2–100%) in rural, and 92.5% (95%CI: 87.8–97.1%) in suburban/urban locations.

### Incorporating healthcare provider perspectives to the beta-version of the tool

Healthcare provider perspectives were used to update the paper-draft and develop a ‘wireframe’ for the beta-version of the tool. Accordingly, we updated the tool to include ‘exercise readiness’, additional conditions (e.g., fatigue), and contextual characteristics such as ‘availability of exercise resources at home’. In this study, a ‘wireframe’ was considered a pdf illustrating the layout, functionality, and content hierarchy for the tool to support a clinical workflow (see Data Supplement, Updated Beta-version of the Clinical Decision Tool (Wireframe)). The ‘wireframe’ included additional components from the Collaborative Deliberation Model [23]. For example, a ‘Decision’ section was added to help the healthcare providers record patient preferences following an exercise discussion (e.g., referral request). The contextual determinants of exercise were linked to resources to help breast cancer survivors learn about ‘at-home exercise’ and ‘healthy eating’.

## Discussion

The ‘American Society of Clinical Oncology (ASCO) Cancer Treatment and Survivorship Care Plan’, now necessary for the national accreditation of breast cancer centers, includes a component for exercise discussions with cancer survivors [38, 39]. However, currently in the U.S., less than 50% of healthcare providers report having discussed exercise with their patients [40] citing lack of confidence, time, and knowledge as barriers [9, 41]. Studies also suggest that healthcare professionals need to be better equipped to engage in exercise discussions in clinical settings [42, 43]. There are several tools including the American College of Sports Medicine’s (ACSM) ‘Moving Through Cancer’ initiative [44] and the ‘Exercise in Cancer Evaluation and Decision Support’ (EXCEEDS) algorithm [45] to help oncology providers assess, advise, and recommend rehabilitation or exercise services to cancer survivors. However, there are limited tools to help providers offer individualized exercise recommendations to breast cancer survivors considering individual demographic, clinical, and contextual characteristics. To our knowledge, this is one of the first studies to specifically assess healthcare provider needs, and characteristics of a clinical decision tool to facilitate individualized exercise recommendations or exercise prescriptions for breast cancer survivors.

In this study, the healthcare providers reported high levels of knowledge on how to encourage patients to exercise. However, the proportion of breast oncologists and primary care physicians knowledgeable about when and how to counsel women about exercise were lower compared to the exercise professionals. They also reported low rates (< 40%) on knowing which patients to refer to an exercise program. These low rates may result from lack of access to screening or surveillance systems for physical inactivity [46], and lack of algorithms to help identify patients for referrals within electronic health records [47, 48]. Studies show that low referrals could increase poor health outcomes, disparities, hospitalizations, and financial burden on healthcare systems [49, 50].

Consistent with previous studies [9, 51], the lowest proportion knowledgeable about how (and when) to counsel women based on current exercise guidelines was seen among advanced care providers, nurses, social workers, and patient navigators. These patterns may be due to insufficient time [52], belief that providing exercise advice is not a part of their role [53], low awareness about clinical exercise guidelines, and doubts about the potential effects of exercise on breast cancer outcomes [9, 51, 54, 55]. Targeted interventions may help improve these healthcare providers’ understanding of the exercise guidelines and their ability to discuss exercise with cancer survivors [56].

Healthcare providers’ confidence may increase the likelihood of exercise discussions in clinical settings [57]. Our results indicate that an evidence-based clinical decision tool providing individualized exercise benefits considering a breast cancer survivor’s individual characteristics, and recommendations based on contextual determinants of exercise may increase healthcare providers’ confidence to engage in exercise discussions. Previous studies also indicate that interventions providing guidance on exercise prescriptions may increase exercise prescription rates [57]. However, the development of such a tool requires multidisciplinary collaborations between breast oncologists, primary care physicians, advanced care providers, exercise specialists, tool developers, mathematical modelers, behavioral and communication scientists, hospital administrators, and importantly diverse breast cancer survivors [26, 28].

Healthcare providers favored a mobile format for the tool. Mobile-based tools may allow patients to gather more information, reflect, and discuss exercise prescriptions prior to and/or following a clinical appointment [58]. A mobile format may also help adapt the tool for electronic health records and support referrals within and across different healthcare systems. However, the tool’s use may vary based on provider type [59]. For instance, in this study, a higher proportion of breast oncologists and primary care physicians reported that they would use the tool to refer patients, whereas exercise specialists reported the tool would be useful to educate, encourage, or facilitate shared decision-making with survivors.

While the sample size for rural providers was limited, the results indicate that provider knowledge on how, when, where, and which patients to counsel or refer may not vary by practice location. Additional research is required to confirm these findings. Interestingly, all practitioners in rural locations reported that a tool to prescribe exercise would be useful in clinical practice. However, a study among rural breast cancer survivors has shown that specific interventions that consider travel distance, access to exercise specialists, and ability to incorporate telehealth networks may be needed to promote exercise in this population [12].

Our results provide important insights to facilitate intervention design, testing, and implementation to increase exercise discussions with breast cancer survivors in clinical settings. Over 90% of the providers agreed that the exercise prescriptions will need to consider ‘exercise readiness’, which reflects the readiness of a survivor to initiate and maintain a behavioral change [60, 61]. Further research is needed to identify ways to efficiently measure and incorporate ‘exercise readiness’ into clinical decision-making. It is important to note that ‘race/ethnicity’ received the lowest rating as an input. Studies suggest that the inclusion of race/ethnicity in clinical decision tools (algorithms) may lead to racism, bias, and discrimination in healthcare [14, 62, 63]. Therefore, in this study, we also asked the providers to rate the inclusion of contextual inputs (e.g., housing, food) that may contribute to the racial and/or ethnic disparities in exercise participation and breast cancer outcomes [20, 64]. The consideration of these factors in a tool may provide a mechanism to address racial and/or ethnic disparities in exercise participation among breast cancer survivors [65].

Our study also had several limitations including a modest sample size to evaluate geographical variability in provider perspectives across practice locations, and the use of self-reported measures for provider/practice characteristics. This limited the ability to explore potential variations in provider knowledge, discussions, and perspectives based on practice characteristics.

### Next steps

We plan to iteratively develop the clinical decision tool in several steps. As described in this study, the first step involved developing the paper-draft and the wireframe (pdf) for the tool with input from healthcare providers. The wireframe will also include perspectives from diverse breast cancer survivors. Next, the updated wireframe (pdf) will be converted to a prototype web-based clinical decision tool for feasibility and acceptability testing. Finally, to inform the implementation of the tool in clinical practice, we will design a clinical trial evaluating the effects of the tool on healthcare providers’: 1) self-efficacy and intentions to engage in conversations about exercise, 2) ability to provide individualized exercise prescriptions and recommendations; and 3) ability to make exercise referrals (when available) for diverse breast cancer survivors.

## Conclusions

Overall, our results suggest that a clinical decision tool that could help provide individualized exercise recommendations and prescriptions may increase provider confidence to discuss, educate, encourage, and refer breast cancer survivors to engage in exercise. Meanwhile, information from this study will inform further development, testing, and implementation of the tool to support individualized exercise prescriptions and discussions with diverse breast cancer survivors.

## Supplementary Material

Supplementary Material

## Figures and Tables

**Table 1:** Healthcare provider and practice characteristics

Characteristics	N	%
Demographic Characteristics		
*Age (n = 177), Median (Standard Deviation (SD), Interquartile Range (IQR))*	43 (16, 34–53)	
*Gender (n = 177)*		
Female	119	67.2
Male	57	32.2
Other	1	0.6
*Race and/or Ethnicity (n = 177)*		
Asian	15	8.5
American Indian/ Alaska Native	5	2.8
Non-Hispanic Black	35	19.8
Hispanic	2	1.1
Native Hawaiian/Pacific Islander	2	1.1
Non-Hispanic White	107	60.5
Mixed	9	5.0
Other	1	0.6
Refused to Answer	1	0.6
Exercise Behavior		
*Aerobic Exercise (n = 177)*		
< 150 moderate-intensity mins/week	42	23.7
≥ 150 moderate-intensity mins/week	131	74.0
Missing	4	2.3
*Muscle-strengthening Exercise (i.e., resistance training) (n = 177)*		
None	2	1.1
< 2 days/week	18	10.2
≥ 2 days/week	144	81.4
Missing	13	7.3
Professional and Training Characteristics		
*Years of Experience (n = 177), Median (SD, IQR) Type of Healthcare Professional*	8 (8, 5–13)	
Breast Oncologists (medical/radiation/surgical)	49	27.7
Primary Care Physicians	19	10.7
Advanced Care Providers (Nurse Practitioners and Physician Assistants)	8	4.5
Patient Navigators/Social Workers/Nurses	34	19.2
Exercise Specialists (Physiologists, Trainers, American College of Sports Medicine (ACSM)/Cancer Exercise Training Institute (CETI)/American Council on Exercise (ACE) Certified)	35	19.8
Occupational/Physical Therapists	32	18.1
*Received Formal Education Regarding Exercise*		
Yes	87	49.1
No	81	45.8
Missing	9	5.1
*Received Continuing Education on Exercise (e.g., Certificate, workshop)*		
Yes	91	51.4
No	84	47.5
Missing	2	1.1
Healthcare Practice Characteristics		
*Practice Setting^1^*		
Community	22	12.4
Municipal Hospital/tertiary care center (inpatient or outpatient)	35	19.8
Private Practice	33	18.6
Academic Institution	29	16.4
National Cancer Institute (NCI) Comprehensive Cancer Center / Cancer Center	67	37.9
Health Maintenance Organization	5	2.8
Other	41	23.2
*U.S. Region*		
Northeast	45	25.4
South	54	30.5
West	58	32.8
Midwest	19	10.7
Missing	1	0.6
*Rural/Urban Status*		
Urban	93	52.5
Suburban	69	39.0
Rural	15	8.5
*Ability to Refer Patients to an Exercise Program*		
No	10	5.7
Yes (within and/or outside the Network)	100	56.5
Missing^2^	67	37.9
Characteristics of Breast Cancer Survivors Seen in Clinical Practice		
*Percentage Treated in Each Stage of Survivorship*		
Pre-treatment*, Median (SD, IQR)*	15 (14.7, 2–30)	
On-treatment*, Median (SD, IQR)*	30 (22.7, 20–50)	
Post-treatment*, Median (SD, IQR)*	25 (22.0, 20–40)	
Palliative/End of Life Care*, Median (SD, IQR)*	10 (12.6, 0–20)	
*Perceived Characteristics of Breast Cancer Survivors*		
Percentage Meeting Aerobic Guidelines*, Median (SD, IQR)*	50 (23.7, 30–70)	
Percentage Meeting Muscle Strengthening Guidelines*, Median (SD, IQR)*	50 (28.3, 20–72)	
Percentage who are Medically Safe to Engage in Exercise*, Median (SD, IQR)*	75 (19.3, 60–90)	
Percentage who are Physically Safe to Engage in Exercise*, Median (SD, IQR)*	74 (21.1, 60–90)	

1 Heatlhcare providers selected multiple categories

2 Exercise specialists did not respond to this question

**Table 2:** Summary of healthcare provider knowledge and discussions about exercise

Characteristics	Total (*N* = 177)	%	Percentage of breast oncologists and primary care physicians reporting Knowledgeable or Likely (*N* = 68)	95% Confidence interval		Percentage of exercise specialists reporting Knowledgeable or Likely (*N* = 67)	95% Confidence interval		Percentage of other healthcare providers^1^ reporting Knowledgeable or Likely (*N* = 42)	95% Confidence interval	
*Knowledge about Counseling*											
Knowledgeable on HOW to Counsel based on Current Exercise Guidelines	110	62.1	55.9	40.1	71.7	86.6	77.8	95.3	33.3	8.6	58.0
Knowledgeable on WHEN to Counsel Patients about Exercise	99	55.9	45.6	28.1	63.1	80.6	70.1	91.1	33.3	8.6	58.0
Knowledgeable on HOW to Encourage Patients to Exercise	141	79.7	72.1	59.5	84.6	91.0	83.9	98.2	73.8	58.3	89.3
*Exercise Discussions*											
Likely to Advise Patients to “Keep Active” DURING Treatment	147	83.1	77.9	66.8	89.1	89.6	81.8	97.3	81.0	67.8	94.1
Likely to Advise Patients to “Keep Active” AFTER Treatment	144	81.4	75.0	63.1	86.9	83.6	73.9	93.3	88.1	77.7	98.5
Likely to Discuss the Role of Exercise in Symptom Management	127	72.2	64.7	50.6	78.8	83.6	73.9	93.3	64.3	46.2	82.4
Likely to Discuss the Role of Exercise in Reducing Recurrence/Death	114	64.4	51.5	34.9	68.0	82.1	72.0	92.2	57.1	37.3	76.9
*Knowledge about Referrals (Excluding Exercise Specialists, Occupational and Physical Therapists) (N = 110)*											
Knowledgeable on WHICH Patients to Refer to a Supervised Exercise Program	39	35.5	39.7	21.3	58.2	-	-	-	28.6	3.0	54.1
Knowledgeable on HOW to Refer Patients to a Supervised/ Unsupervised Exercise Program	78	70.9	75.0	63.1	86.9	-	-	-	64.3	46.2	82.4
Knowledgeable on WHO and WHERE to Refer Patients	55	50.0	69.1	55.9	82.3	-	-	-	61.9	43.2	80.6

1 Advanced Care Providers/Patient Navigators/Social Workers/Nurses

**Table 3:** Healthcare provider perspectives on a clinical decision tool for individualized exercise prescriptions

Characteristics	Total (*N* = 177)	%	Percentage of breast oncologists and primary care physicians reporting ‘Agreement’ (*N* = 68)	95% Confidence interval		Percentage of exercise specialists reporting ‘Agreement’ (*N* = 67)	95% Confidence interval		Percentage of other^1^ reporting ‘Agreement’ (*N* = 42)	95% Confidence interval	
*Usefulness*											
Healthcare Provider Finds such a Tool Useful	148	83.6	72.1	59.5	84.6	77.6	66.3	88.9	92.9	84.8	100.0
Healthcare Provider would Use Tool on a Regular Basis	146	82.5	79.4	68.6	90.2	88.1	79.8	96.3	78.6	64.6	92.6
Tool Increases Healthcare Provider’s Confidence	149	84.2	83.8	74.3	93.4	89.6	81.8	97.3	95.2	88.6	100.0
*Format*											
Mobile Application	154	87.0	86.8	78.1	95.4	80.6	70.0	91.1	97.6	93.0	100.0
Web-based tool integrated with Electronic Health Records	140	79.1	76.5	64.9	88.0	77.6	66.3	88.9	85.7	74.3	97.1
Paper-based tool	100	56.5	58.8	43.6	74.1	61.2	46.3	76.1	45.2	22.9	67.6
*Timing*											
Before a Clinical Consultation (patients to use on their own)	100	56.8	73.1	60.7	85.5	41.8	23.5	60.1	54.8	34.4	75.1
Before a Clinical Consultation (with a care coordinator or a patient navigator)	110	62.9	73.5	61.3	85.8	54.5	38.3	70.8	58.5	38.8	78.2
During a Clinical Consultation (with a healthcare provider)	142	80.7	80.9	70.5	91.3	83.6	73.9	93.3	75.6	60.5	90.7
After a Clinical Consultation (patients to use on their own)	131	75.3	72.1	59.5	84.6	67.7	53.9	81.5	92.7	84.4	100.0
After a Clinical Consultation (with a care coordinator or a patient navigator)	128	73.6	61.8	47.1	76.5	76.9	65.2	88.6	87.8	77.1	98.5
*Uses (Only among Healthcare Providers who “Agreed” the Tool would be Useful in Clinical Practice (N = 148))^2^*											
Educate Patients	131	88.5	85.7	75.1	96.3	91.7	84.4	99.0	87.2	75.9	98.4
Encourage Patients to Exercise	136	91.9	87.8	78.0	97.6	93.3	86.8	99.9	94.9	87.8	100.0
Facilitate Shared Decision-Making about Exercise	123	83.1	69.4	53.9	84.9	91.7	84.4	99.0	87.2	75.9	98.4
Identify Resources to Support Exercise	119	80.4	75.5	61.7	89.4	83.3	73.0	93.7	82.1	68.8	95.3
Refer Patients to Exercise Professionals	125	85.0	87.8	78.0	97.6	86.4	77.0	95.8	79.5	65.3	93.7

1 Advanced Care Providers/Patient Navigators/Social Workers/Nurses

2 Healthcare providers who “Agreed” that the tool will be useful in clinical practice (*N* = 148) included 49 clinicians, 60 exercise specialists, and 39 advanced care providers, nurses, patient navigators, and social workers

## Data Availability

No datasets were generated or analysed during the current study.
