# Supplementary material for: Healthcare provider perspectives on a clinical decision tool to support individualized exercise prescriptions and discussions for breast cancer survivors
Source: J Cancer Surviv. 2025 Mar 12;20(4):1499–512. doi: 10.1007/s11764-025-01750-3 (PMC12310178; doi:10.1007/s11764-025-01750-3)
Supplement: Supplementary file 1 — Supplementary file1 (PDF 2835 KB) [file 11764_2025_1750_MOESM1_ESM.pdf]

Supplementary Material

Healthcare Provider Perspectives on a Clinical Decision Tool to Support Individualized Exercise Prescriptions and Discussions for Breast Cancer Survivors

Data Supplement: Survey Items

Measure	Categories	Source
Type of healthcare profession		Clinicians' Perspectives on Exercise in Patients with Cancer (CliPEC) questionnaire[1]
Race, Ethnicity		National Institutes of Health (NIH) Common Data Elements (CDE) repository[2]
Exercise Participation	1. Aerobic exercise (min/week)<br>2. Muscle-strengthening exercise (days/week)	Health Information National Trends Survey (HINTS)[3]
Type of practice	Private practice, academic institution, non-profit organization, and more	Ramsey et al.[4]
Rural/urban location	Urban, suburban, rural	Powers et al.[5]
Ability to provide a referral	No, yes – within healthcare network, yes – outside of healthcare network	
Current practices, knowledge, barriers, and facilitators for providing exercise advice	Five-point scale ranging from 1 (very unlikely) to 5 (very likely); 1 (no knowledge) to 5 (very knowledgeable); 1 (strongly disagree) to 5 (strongly agree)	Clinicians' Perspectives on Exercise in Patients with Cancer (CliPEC) questionnaire[1]
Timing	Before a clinical consultation (on their own); before a clinical consultation (with a care coordinator or patient navigator); during a clinical consultation with the health care provider; after clinical consultation (on their own); after a clinical consultation (with a care coordinator or patient navigator)	O'Neill et al. 2018.[6]
Tool Characteristics	**Modality:** paper, web-based integrated with electronic health records, and mobile application<br>**Individual and clinical factors:** age, race and ethnicity, stage at diagnosis, tumor characteristics, current treatment (e.g., chemotherapy, radiotherapy, endocrine therapy, surgery), and historical treatments<br>**General health and exercise preferences:** current comorbidities, physical impairments (e.g., mobility, reaching, etc.), functional impairments (e.g., challenges with daily living), cognitive impairments (e.g., changes in memory or attention, difficulty problem solving, etc.), psychological impairments (e.g., changes in mood, feeling down, etc.), patient personal preferences (e.g., what activities they enjoy doing), and readiness to exercise<br>**Contextual factors:** childcare (child supervision); clothing (availability of	NIH CDE repository[2] and Cognitive Interview

Measure	Categories	Source
	appropriate clothing and shoes); finances (problems with insurance or debt), food (access to healthy food), housing (availability of safe and secure housing), internet/broadband (availability of internet to check email and schedule exercise sessions), phone (e.g., mobile or landline; availability of a phone to connect with peer and health care professional), transportation (availability of transportation), utilities (whether an individual had utility companies cut off service due to not paying bills), residential greenness (availability of green spaces), neighborhood safety (safety of local environment), access to a facility or exercise program in the neighborhood or at work, and access to exercise resources at home	
Individualized Benefits	Improved quality of life, more likely to stay cancer recurrence free, less likely to die of breast cancer, less likely to die of cardiovascular disease, less likely to die of all causes, improved life expectancy, less tiredness / more energy, improved cognition, greater ability to do everyday tasks, less likely to experience anxiety, less likely to experience depressive symptoms, less likely to experience lymphedema, improved bone health, improved sleep, less likely to be admitted to the hospital, less like to experience adverse side effects during treatment, and other (open ended question)	Campbell et al.[7] and Cognitive Interview
Conditions associated with cancer and cancer treatment	Cognitive difficulty (mental slowing), neuropathy, sarcopenia/muscle weakness, slowing and fatigue, bone loss, bone metastases, lymphedema, symptom clusters, arthritis/musculoskeletal issues, ataxia, severe nutritional deficiencies, cardiopulmonary disease, nausea or diarrhea, sexual dysfunction, and other (open ended question)	Campbell et al.[7] and Cognitive Interview
Outputs	Personalized benefits and risks for aerobic exercise, personalized benefits and risks for muscle-strengthening exercise, printable handouts of benefits and risks of exercise, exercise referral suggestions based on patient preferences	

Supplemental Table (Education): Education Received Regarding Physical Activity and/or Exercise

Theme (Total N=177)	N	%
**Formal education (i.e., as part of a degree)**		
Some	85	48.0
Exercise science/kinesiology	36	20.3
Physical therapy	19	10.7
Medicine	13	7.3
Nursing	9	5.1
Occupational therapy	6	3.4
Physical education	5	2.8
Nutrition	2	1.1
Athletic training	1	0.6
Public health	1	0.6
None/Minimal	83	46.9
Missing	9	5.1
**Continuing education (i.e., as part of a certification or workshop)**		
Some	89	50.3
Cancer exercise trainer/specialist certifications*	35	19.8
Conference course / Workshops / Continuing Education Units	28	15.8
Personal trainer certifications	14	7.9
Clinical Exercise Physiologist certification	8	4.5
Certified Lymphedema Therapy certification	6	3.5
Certified Strength and Conditioning Specialist	6	3.5
Yoga / Pilates certifications	5	2.8
None/Minimal	86	48.6
Missing	2	1.1
Formal and continuing	58	32.8

Note. Healthcare providers reported more than one form of formal and/or continuing education.
*Cancer Exercise Training Institute (CETI) Exercise Specialist ®, Klose Training Strength After Breast Cancer, Maple Tree Certified Exercise Oncology Instructor, American Council on Exercise (ACE) Cancer Exercise Specialist, or University of Northern Colorado Cancer Rehabilitation Institute (UNCCRI) Clinical Cancer Exercise Specialist

Supplemental Table (Resources). Resources or Tools Currently used by Healthcare Providers to Discuss Exercise with Breast Cancer Survivors

Theme (Total N=177)	N	%
None	59	33.3
Educational material	39	22.0
Written	22	12.4
Digital	17	9.6
Unclear Format	11	6.2
Guidelines	15	8.5
Research/publications	2	1.1
Self-developed	4	2.3
Exercise log	1	0.6
Fitness tracker	4	2.3
Mobile/web application	10	5.6
Software	5	2.8
Video	3	1.7
Website	8	4.5
Telehealth platform	3	1.7
Workshop/webinar/course	3	1.7
Referral (explicit)	8	4.5
Referral (implied)	9	5.1
Missing	20	11.3

Note. Providers reported one or more resources or tools used to discuss exercise with breast cancer survivors.

Supplemental Figure (Beta version of the Clinical Decision Tool): Screenshots of the Beta Version (Paper-draft) of the Clinical Decision Tool for Individualized Exercise Prescriptions and Discussions Shown to the Healthcare Providers

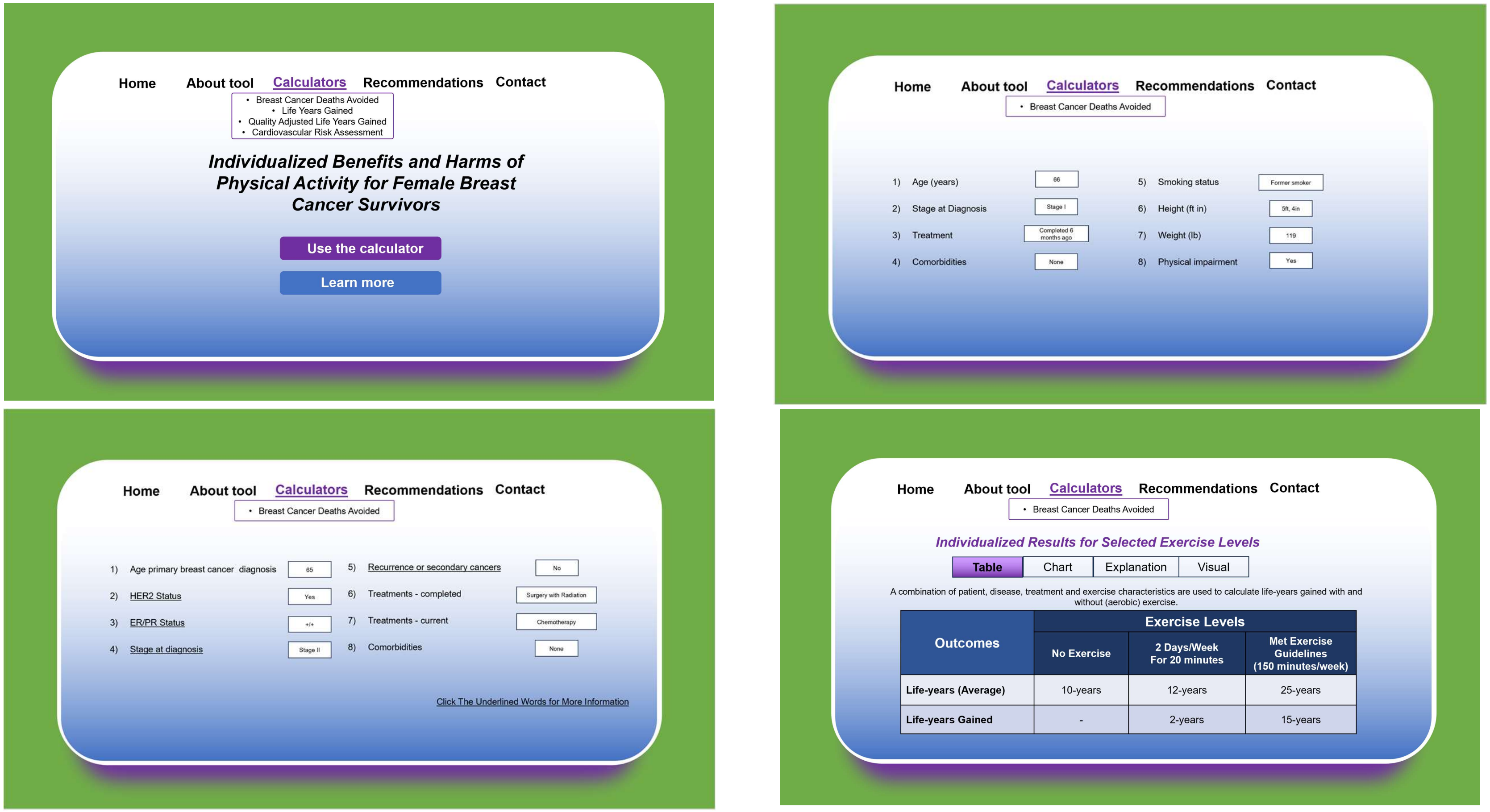

Supplemental Table (Additional Feedback): Additional Feedback on the Clinical Decision Tool

Theme	N	%
**Contribute to Confidence (N=87)**		
Access to evidence-based information	33	37.9
Discussion guidance	22	25.3
Individualized	18	20.7
Increase healthcare provider knowledge	8	9.2
Patient autonomy	8	9.2
Accessibility	6	6.9
Patient education	6	6.9
Interprofessional communication	6	6.9
Patient engagement	5	5.7
Generation of patient handout	4	4.6
Clinical workflow efficiency	4	4.6
Easy to use	3	3.4
Recommendation standardization	2	2.3
Referral guide	1	1.1
**More Useful (N=40)**		
Community forum / interactive content	14	35.0
Educational resources	9	22.5
Feedback / suggestions section / live support	7	17.5
Language options / conversation guides	4	10.0
More individualization features	4	10.0
Accessibility	4	10.0
Electronic health record linkage for automatic filling	3	7.5
Success stories	3	7.5
Patient autonomy	2	5.0
Safety and risk management	1	2.5
Privacy	1	2.5
**Change to Increase Confidence (N=15)**		
Patient Education Material	3	20.0
Increased Patient Accessibility	3	20.0
Virtual coaching / interactive support	3	20.0
Electronic health record integration	2	13.3
Gamification	2	13.3
Structured scripts	1	6.7
Interprofessional collaboration	1	6.7

Note. Percentages were calculated based on the number of relevant responses to respective open-ended questions

Supplemental Table (Knowledge and Discussions by Practice Location): Comparison of Healthcare Provider Knowledge and Exercise Discussions by Healthcare Provider Practice Location

Characteristics	Percentage of Healthcare Providers[1] Practicing in Urban/Suburban Locations Reporting Knowledgeable or Likely (N=162)	95% Confidence Interval		Percentage of Healthcare Providers[2] Practicing in Rural Locations Reporting Knowledgeable or Likely (N=15)	95% Confidence Interval	
**Knowledge about Counseling**						
Knowledgeable on HOW to Counsel based on Current Exercise Guidelines	62.3%	52.9%	71.8%	60.0%	28.0%	92.0%
Knowledgeable on WHEN to Counsel Patients about Exercise	57.4%	47.4%	67.5%	40.0%	0.8%	79.2%
Knowledgeable on HOW to Encourage Patients to Exercise	78.4%	71.2%	85.6%	93.3%	80.3%	100.0%
**Exercise Discussions**						
Likely to Advise Patients to "Keep Active" DURING Treatment	84.0%	77.8%	90.1%	73.3%	47.2%	99.5%
Likely to Advise Patients to "Keep Active" AFTER Treatment	81.5%	74.9%	88.1%	80.0%	57.4%	100.0%
Likely to Discuss the Role of Exercise in Symptom Management	72.0%	63.9%	80.2%	73.3%	47.2%	99.5%
Likely to Discuss the Role of Exercise in Reducing Recurrence/Death	63.6%	54.3%	72.9%	73.3%	47.2%	99.5%
**Knowledge about Referrals (Excluding Exercise Specialists, Occupational and Physical Therapists) (N=110)**						
Knowledgeable on WHICH Patients to Refer to a Supervised Exercise Program	35.9%	20.5%	51.4%	33.3%	0.0%	98.7%
Knowledgeable on HOW to Refer Patients to a Supervised/ Unsupervised Exercise Program	54.4%	41.3%	67.4%	33.3%	0.0%	74.7%
Knowledgeable on WHO and WHERE to Refer Patients to	57.3%	44.7%	69.9%	66.7%	20.5%	100.0%

[1]Healthcare providers practicing in the Urban/Suburban locations included breast oncologists/ primary care physicians (n=67, 41.4%); exercise specialists and occupational/physical therapists (n=59, 36.4%); and advanced care providers, nurses, social workers, and patient navigators (n=36, 22.2%)
[2]Healthcare providers practicing in the Rural locations included breast oncologists/primary care physicians, exercise specialists and occupational/physical therapists (n=9, 60.0%); and advanced care providers, nurses, social workers, and patient navigators (n=6, 40.0%)

Supplemental Table (Tool Perspectives by Practice Location): Comparison of Perspectives on a Personalized Clinical Decision Tool for Exercise Prescriptions by Healthcare Provider Practice Location

Characteristics	Percentage of Healthcare Providers[1] Practicing in Urban/Suburban Locations Reporting Agreement (N=162)	95% Confidence Interval		Percentage of Healthcare Providers[2] Practicing in Rural Locations Reporting Agreement (N=15)	95% Confidence Interval	
**Usefulness**						
Healthcare Provider Finds Such a Tool Useful	82.1%	75.6%	88.6%	100.0%	100.0%	100.0%
Healthcare Provider would Use Tool on a Regular Basis	82.1%	75.6%	88.6%	86.7%	68.2%	100.0%
Tool Increases Healthcare Provider's Confidence	84.0%	77.8%	90.1%	86.7%	68.2%	100.0%
**Format**						
Mobile Application	88.3%	83.0%	93.5%	73.3%	47.2%	99.5%
Web-based integrated with Electronic Health Records	79.6%	72.7%	86.6%	73.3%	47.2%	99.5%
Paper-based	54.9%	44.6%	65.3%	73.3%	47.2%	99.5%
**Timing**						
During a Clinical Consultation (with a healthcare provider)	81.4%	74.7%	88.0%	73.3%	47.2%	99.5%
Before a Clinical Consultation (with a care coordinator or a patient navigator)	64.0%	54.7%	73.2%	50.0%	13.0%	87.0%
Before a Clinical Consultation (patients to use on their own)	58.4%	48.4%	68.3%	40.0%	0.8%	79.2%
After a Clinical Consultation (patients to use on their own)	75.5%	67.8%	83.2%	73.3%	47.2%	99.5%
After a Clinical Consultation (with a care coordinator or a patient navigator)	74.2%	66.3%	82.1%	66.7%	37.4%	95.9%

Characteristics	Percentage of Healthcare Providers[1] Practicing in Urban/Suburban Locations Reporting Agreement (N=162)	95% Confidence Interval		Percentage of Healthcare Providers[2] Practicing in Rural Locations Reporting Agreement (N=15)	95% Confidence Interval	
**Uses (Only among Healthcare Providers who "Agreed" the Tool would be Useful in Clinical Practice (N=148))[3]**						
Encourage Patients to Exercise	92.5%	87.8%	97.1%	86.7%	68.2%	100.0%
Refer Patients to Exercise Professionals	88.6%	82.9%	94.4%	53.3%	18.8%	87.9%
Educate Patients	90.2%	84.9%	95.5%	73.3%	47.2%	99.5%
Identify Resources to Support Exercise	82.7%	75.6%	89.8%	60.0%	28.0%	92.0%
Facilitate Shared Decision-Making	84.2%	77.5%	91.0%	73.3%	47.2%	99.5%

[1]Healthcare providers practicing in the Urban/Suburban locations included breast oncologists/ primary care physicians (n=67, 41.4%); exercise specialists and occupational/physical therapists (n=59, 36.4%); and advanced care providers, nurses, social workers, and patient navigators (n=36, 22.2%)
[2]Healthcare providers practicing in the Rural locations included breast oncologists/primary care physicians, exercise specialists and occupational/physical therapists (n=9, 60.0%); and advanced care providers, nurses, social workers, and patient navigators (n=6, 40.0%)
[3]Only healthcare providers who "agreed" that the tool will be useful in clinical practice were asked about the uses of the tool. This included 49 clinicians, 60 exercise specialists, and 39 advanced care providers, nurses, patient navigators, and social workers

Supplemental Figure: Updated Beta-version of the Clinical Decision Tool (Wireframe)

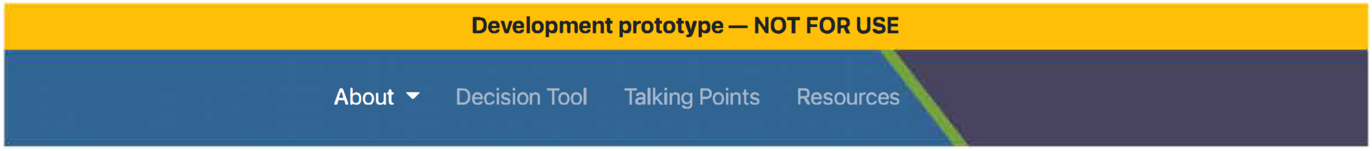

Exercise:

- Can reduce the risk of chronic diseases such as cardiovascular disease, diabetes, and osteoporosis,
- Can reduce common side effects of breast cancer treatment,
- May reduce the risk of the cancer coming back and improve survival.

This tool can help you:

- Discuss individualized benefits (and harms) of different levels of exercise,
- Develop individualized talking points to promote exercise, and
- Identify online resources for exercise promotion and education.

How to Use the Tool

A Decision Tool to Provide the Individualized Benefits of Exercise

The Decision Tool Calculators use information on the breast cancer survivor's age, stage at diagnosis, hormone receptor status, HER-2 (human epidermal growth factor receptor) status, completed treatment, weight, height, and other health conditions (see health questions) to provide individualized health outcomes associated with exercise.

The individualized outcomes include quality-of-life, 10-year risk of breast cancer death, and life-years gained associated with different levels of exercise based on the breast cancer survivor's individual characteristics.

Health questions related to access to exercise resources at home, access to healthy food, and transportation links to additional resources to help discuss barriers to exercise.

The decision tab of the tool is designed to help breast cancer survivors and their health care providers assess their preferences for next steps regarding exercise.

The information on outcomes of exercise is derived from mathematical models created by the team led by Dr. Jinani Jayasekera at the Health Equity and Decision Sciences research laboratory at NIH. Please see the Scientific Basis page for more information.

Counseling

Health Care Professional Talking Points

This section of the site provides talking points and strategies for health care professionals to engage breast cancer survivors in shared decision-making about exercise.

EMR Documentation

This site provides example text that may be cut and pasted into electronic medical records to help document conversations about exercise with breast cancer survivors.

Use the Decision Tool

Development prototype — NOT FOR USE

About Decision Tool Talking Points Resources

Exercise Readiness Health Information Conditions Exercise Behavior Health Outcomes Decision Context Summary

Exercise Readiness

Next

Please read the questions below carefully and be ready to discuss the answers with your healthcare provider

Do you know of any reason why you should not exercise?

- [ ] Yes
- [x] No
- [ ] Don't know

Breast Cancer Diagnosis

Next

Fill With Random Data Clear Data

How old were you when diagnosed with breast cancer (in years)?

55

What was your breast cancer stage at diagnosis?

I

What was your breast cancer HER2 status?

Negative

What was your breast cancer ER/PR status?

+/-

Have you had breast cancer recurrence or secondary cancer?

No

Which treatments have already been completed?

- [x] Surgery (Lumpectomy or Mastectomy)
- [ ] Radiation
- [ ] Chemotherapy
- [ ] Hormone (endocrine) Therapy
- [ ] Immunotherapy (biologicals)

Which treatments are currently ongoing?

- [ ] Surgery (Lumpectomy or Mastectomy)
- [ ] Radiation
- [ ] Chemotherapy
- [x] Hormone (endocrine) Therapy
- [ ] Immunotherapy (biologicals)

Development prototype — NOT FOR USE

About Decision Tool Talking Points Resources

Exercise Readiness | **Health Information** | Conditions | Exercise Behavior | Health Outcomes | Decision | Context | Summary

Other Health Information

Next

Do you currently smoke?

No

Do you currently drink alcohol?

Yes

How tall are you (in inches)?

64

How much do you weigh (in pounds)?

198

Previous Next

Development prototype — NOT FOR USE

About | Decision Tool | Talking Points | Resources

Exercise Readiness | Health Information | **Conditions** | Exercise Behavior | Health Outcomes | Decision | Context | Summary

Conditions

Next

What other conditions do you have?

- [x] Diabetes
- [ ] Peripheral Vascular Disease
- [ ] Paralysis
- [ ] Stroke
- [ ] COPD/emphysema
- [ ] Congestive heart failure (CHF)
- [ ] Chronic kidney disease or on dialysis
- [ ] Moderate or severe liver disease or cirrhosis
- [ ] Dementia
- [ ] Aortic Aneurysm
- [ ] Peripheral Neuropathy
- [ ] Arthritis/Musculoskeletal Issues
- [ ] Poor bone health
- [ ] Lymphedema
- [ ] Lung or abdominal surgery
- [ ] Ostomy
- [ ] Cardiopulmonary Disease
- [ ] Ataxia
- [ ] Extreme fatigue
- [ ] Severe nutritional deficiency
- [ ] Worsening/changing physical condition
- [ ] Bone metastases
- [ ] None

Next

Development prototype — NOT FOR USE

About Decision Tool Talking Points Resources

Exercise Readiness Health Information Conditions **Exercise Behavior** Health Outcomes Decision Context Summary

Exercise Behavior

Next

In a typical week, how many days do you exercise (please do not include weightlifting)?

1

On the days that you do any exercise, how long do you typically do these activities?

15

In a typical week, outside of your job or work around the house, how many days do you exercise to strengthen your muscles?

1

Previous Next

Development prototype — NOT FOR USE

About Decision Tool Talking Points Resources

Exercise Readiness Health Information Conditions Exercise Behavior **Health Outcomes** Decision Context Summary

Breast Cancer Survival

Next

Breast Cancer Outcomes	Mathematically Modeled Estimates for 10-year Breast Cancer Specific Survival (%)		
	**Current Level of Exercise (No/Minimal Exercise)**	**30 to 60 minutes of Aerobic Exercise (Less than Meeting Exercise Guidelines)**	**Meeting Exercise Guidelines**
**Breast Cancer-Specific Survival**	86%	88%	91%
**Absolute Difference**	-	2%	5%

"*Meeting Exercise Guidelines*" is engaging in the equivalent of ≥150 minutes per week of moderate intensity aerobic physical activity (e.g., walking running, cycling); and ≥2 days per week of muscle strengthening exercise.

For women with these individual characteristics:

- Breast cancer-specific survival with approximately your current level of exercise is 86%.
- Breast cancer-specific survival when active **(**i.e. meeting exercise guidelines**)** is 91%.
  - Thus, meeting exercise guidelines provides an average additional gain of 5%.

Previous Next

Development prototype — NOT FOR USE

About Decision Tool Talking Points Resources

Exercise Readiness Health Information Conditions Exercise Behavior Health Outcomes **Decision** Context Summary

Decision

Next

At this point, what are you thinking about exercise?

Wants to increase exercise; requests a referral

Previous

Next

Development prototype — NOT FOR USE

About Decision Tool Talking Points Resources

Exercise Readiness | Health Information | Conditions | Exercise Behavior | Health Outcomes | Decision | **Context** | Summary

Context

Next

Do you have exercise equipment at home?

No

Please click here for information about exercise you can do at home

Do you need additional information about eating healthy? Please click the links below,

- [ ] Local Food Resources
- [ ] Healthy Food Recipes
- [x] Meal Prep Services
- [ ] Talk to a Dietician
- [ ] Other

Do you need information about transportation services available in your area?

Yes

Previous

Next

References

1. Nadler MB, Bainbridge D, Fong AJ, Sussman J, Tomasone JR, Neil-Sztramko SE. Moving Cancer Care Ontario's Exercise for People with Cancer guidelines into oncology practice: using the Theoretical Domains Framework to validate a questionnaire. *Support Care Cancer*. Jun 2019;27(6):1965-1968. doi:10.1007/s00520-019-04689-1
2. National Institutes of Health Data Management and Sharing Policy. National Institutes of Health. Accessed September 16, 2024. https://cde.nlm.nih.gov/home
3. National Cancer Insititute. Health Information National Trends Survey. National Cancer Insititute. Accessed September 16, 2024. https://hints.cancer.gov/data/default.aspx
4. Ramsey I, Chan A, Charalambous A, et al. Exercise counselling and referral in cancer care: an international scoping survey of health care practitioners' knowledge, practices, barriers, and facilitators. *Supportive Care in Cancer*. 2022/11/01 2022;30(11):9379-9391. doi:10.1007/s00520-022-07342-6
5. Powers SL, Pitas NA, Mowen AJ. Critical consciousness of systemic racism in parks among park agency directors and policymakers: An environmental justice tool for recreation and conservation leaders. *Soc Nat Resour*. 2023:1-24. doi:10.1080/08941920.2023.2250737
6. O'Neill SC, Taylor KL, Clapp J, et al. Multilevel influences on patient-oncologist communication about genomic test results: Oncologist perspectives. *Journal of Health Communication*. 2018/07/03 2018;23(7):679-686. doi:10.1080/10810730.2018.1506836
7. Campbell KL, Winters-Stone KM, Wiskemann J, et al. Exercise Guidelines for Cancer Survivors: Consensus Statement from International Multidisciplinary Roundtable. *Med Sci Sports Exerc*. Nov 2019;51(11):2375-2390. doi:10.1249/mss.0000000000002116
